# Vitamin D receptor gene polymorphism and its association with the susceptibility to *Helicobacter pylori* infection in the Egyptian population with hepatocellular carcinoma

**DOI:** 10.1186/s12876-026-04740-6

**Published:** 2026-04-02

**Authors:** Asmaa Ibrahim, Shaymaa Abdelraheem Abdelhady, Fatma Rageh, Rasha Elgamal, Mohamed Medhat, Reham F. Othman, Hend A. Yassin, Yasmine N. Kamel, Eman M. Osman, Almaza Ali Salim, Samar S. Ahmed, Reham Mohamed Shaker, Doaa Eltaweel

**Affiliations:** 1 Genetic Engineering and Biotechnology Research Institute, University of Sadat City (GEBRI, USC), Sadat, Egypt; 2 Department of Medical Parasitology, Faculty of Medicine, Cairo University (Laboratory of Molecular Medical Parasitology, LMMP), Cairo, Egypt; 3 Department of Clinical Pathology, Suez Canal University, Suez, Egypt; 4 Department of Infectious Diseases, Gastroenterology, and Hepatology, Faculty of Medicine, Suez University, Suez, Egypt; 5 Department of Clinical Pathology, Faculty of Medicine, Suez University, Suez, Egypt; 6 Department of Medicine, School of Medicine, Newgiza University, Giza, Egypt; 7 Department of Medical Biochemistry, Faculty of Medicine, Alexandria University, Alexandria, Egypt; 8 Department of Immunology and Allergy, Medical Research Institute, Alexandria University, Alexandria, Egypt; 9 Department of Family Medicine, Faculty of Medicine, Port Said University, Port Said, Egypt; 10 Department of Public Health and Occupational Medicine, Faculty of Medicine, Suez University, Suez, Egypt; 11 Department of Nephrology, Faculty of Medicine, Suez University, Suez, Egypt; 12 Department of Medical Microbiology and Immunology, Faculty of Medicine, Cairo University, Cairo, Egypt

**Keywords:** *Helicobacter pylori*, Liver diseases, Vitamin D receptor, Polymorphism, Egyptian, Susceptibility

## Abstract

**Objective:**

This study aimed to estimate the frequency of *H. pylori* infection in patients with liver disorders, and identify the relationship between vitamin D receptor gene variants and susceptibility to *H. pylori* infections and HCC in Egyptian patients with liver diseases.

**Materials and methods:**

This study consists of 300 adult patients classified into three groups: 100 healthy controls, 100 patients with liver cirrhosis, and 100 patients with HCC. Every patient was assessed for the presence of *H. pylori* by rapid test and polymerase chain reaction (PCR). Estimation of *FokI* and *BsmI* VDR gene polymorphism was performed by restriction fragment length polymorphism-Polymerase chain reaction (RFLP-PCR).

**Results:**

Infection with *H. pylori* was present in 46.7% of cases overall. Liver cirrhosis (LC) patients had a higher prevalence of *H. pylori* infection, followed by HCC patients (59% and 42%, respectively). Patients with LC and HCC were shown to be *CagA* positive, with the target *CagA* oncogene gene having an expected product size of 37.3% and 35.7%, respectively. LC and HCC patients showed a significant difference between the *H. pylori*-positive and -negative groups. Concerning the BsmI polymorphism, HCC patients with *H. pylori-CagA* positive had a higher GC genotype than those with *H. pylori-CagA* negative, and LC and HCC patients with *H. pylori-CagA* positive had a higher TT genotype than those with *H. pylori-CagA* negative.

**Conclusion:**

*FokI* and *BsmI* VDR polymorphisms may be linked to *H. pylori* infection and *CagA* strain susceptibility in patients with LC and HCC.

## Introduction

A common bacterial pathogen worldwide, *Helicobacter pylori (H. pylori)* usually colonises the mucosal tissue of the stomach. *H. pylori* is believed to be prevalent in over 50% of individuals worldwide. *H.pylori* demographics and geographic locations influence pylori infection rates; countries with low incomes typically have higher infection rates than high-income ones [1]. In Egypt has a very high prevalence of *H. pylori* infections, with studies reporting rates as high as 70% in the general population, even reaching over 80–90% in specific patient groups like those with chronic Hepatitis C (HCV) [2–4]. The World Health Organisation (WHO) has designated *H. pylori* as a class I carcinogen. Among several factors that make *H. pylori* pathogenic are the virulence genes *CagA* and *VacA*, which are implicated in colonisation, chronic inflammation, and carcinogenic processes [5].

Hepatocellular carcinoma (HCC) is a serious global public health hazard that can result from liver cirrhosis [6]. In Egypt, HCC is a significant public health problem [7]. The incidence of HCC has approximately doubled in Egypt over the last decade, and it represents the main complication of cirrhosis [8, 9]. A significant association was observed between infection with *H. pylori*, elevated titers of *H. pylori* antibodies, and an increased risk of HCC in Egyptian patients [10]. *H. pylori* infections can directly exacerbate inflammatory stomach lesions in individuals with liver cirrhosis and indirectly cause liver function disorders [6].

One of the most important micronutrients in the human body, vitamin D is primarily responsible for calcium homeostasis and bone mineralisation. The synthesis of calcitriol, the active form of vitamin D, which binds to vitamin D receptors (VDRs), requires two hydroxylations that are produced by the liver and kidneys. Vitamin D thus controls several biological functions [11]. Many immune cell types, such as lymphocytes, monocytes, macrophages, and dendritic cells, express the VDR and metabolising enzymes [12]. Supplementing with vitamin D appears to prevent systemic infections, respiratory, digestive, and urinary tract infections, which have been associated with low vitamin D levels [13, 14].

One of the possible risk factors for the failure of *H. pylori* treatment is vitamin D deficiency; studies suggest supplementing with this vitamin as a supplement to regular therapy [15]. Additionally, 90% of individuals with HCC have vitamin D insufficiency, which is an epiphenomenon in the context of advanced-stage concurrent liver disease (ACLD). In the context of HCC, vitamin D’s anticancer qualities have been shown in recent years [16]. The 12q13.11 chromosome contains the VDR gene, which is more than 60 kb in length. The FokI, BsmI, ApaI, and TaqI restriction enzymes’ restriction fragment length polymorphism (RFLP) is used to examine both treatment response and genetic susceptibility to infectious diseases [17]. Additionally, in a number of populations, genetic polymorphisms within VDR are linked to an increased risk of HCC [18, 19].

Consequently, an association between vitamin D and *H. pylori* infection has been discovered, beginning with a correlation between vitamin D deficiency and various types of infections. This finding warrants additional investigation. This study focused on determining the incidence of *H. pylori* infection in liver disease patients and the relationship between vitamin D receptor gene variants and *H. pylori* infection and HCC risk in liver disease patients from Egypt.

### Study design and populations

Three hundred patients who attended the Tropical Medicine department at Suez University Hospitals in Egypt participated in this case-control study. The participants were divided into three groups: 100 healthy individuals who had no history of liver disease; 100 patients with chronic liver cirrhosis (LC) from hepatitis C; and 100 patients with cirrhosis from hepatitis C who had hepatocellular carcinoma (HCC). HCV patients had either received a diagnosis or were receiving follow-up care. Through the use of ELISA and real-time PCR testing, anti-HCV antibodies and HCV RNA were used to confirm the HCV infection. Along with abdominal US and spiral CT imaging, serum alpha-fetoprotein (AFP) was used to diagnose HCC patients [20].

Patients were diagnosed with liver cirrhosis (LC) based on a combination of radiological, laboratory, and clinical data. This study excluded participants with autoimmune diseases and HIV or HBV. Additionally, the study excluded patients who were pregnant, abused alcohol, used illegal drugs, reported taking *H. pylori* medication within the past six months, or had received a proton pump inhibitor (PPI) from a subject within the preceding month. Use of antibiotics, immunosuppressive treatment, anti-inflammatory drugs, or corticosteroids during the previous two months. Cancers, renal insufficiency, and gastric surgery were excluded.

The local ethics committee of Suez Canal University approved the current study (IRB No. Research 5947#). We verified that all study procedures follow the appropriate local regulatory regulations and the principles of the most recent version of the Declaration of Helsinki [21]. Before the collection of demographic information and blood samples, each participant provided verbal informed consent after being briefed on the study’s objectives.

### Samples collection and laboratory investigations

For every patient, 8 millilitres of venous blood were drawn. After collecting 4mL were collected of the sample in a plain vacutainer tube. The tube was left to clot at room temperature, which usually takes about 20 min. Then the serum was separated by centrifugation at 2000–3000 rpm for 5 min. Serum was used for measurement of Liver functions, kidney functions, lipid profile, glucose profile, and electrolytes following the guidelines provided by the kit’s manufacturer by a fully automated auto-analyzer Cobas c 6000 (“Roche Diagnostics, Mannheim, Germany”). Part of the serum was stored at -20 °C for further analysis of Serum anti-*H. pylori* antibodies. Four milliliters were collected in an ethylene-diamine-tetraacetic acid (EDTA) vacutainer tube, 2 mL for complete blood count done by Sysmex 5 differential part (Siemens AG, Erlangen, Germany), and 2 mLfor extraction of genomic DNA and molecular analysis.

### Detection of *H.pylori* antibodies

Serum anti-*H. pylori* antibodies were detected using the immunochromatographic OnSite^®^ *H. pylori* Ab Combo Rapid Test (CTK Biotech, San Diego, CA1, USA, cat.no. R0191C.)

### Molecular analysis

#### DNA isolation

The Qiagen blood kit was used to extract genomic DNA in accordance with the manufacturer’s instructions. The DNA’s integrity was shown using the 1% agarose gel. DNA purity and concentration were evaluated using NanoDropTM 2000/2000c (Thermo Fisher Scientific, Waltham, MA, USA). For SNP genotyping and detecting *H. pylori*, the isolated DNA is stored at -20 °C for further analysis.

### Molecular identification and detection of *H. pylori*

Nested PCR (n-PCR) for *H. pylori* targeting the UreA gene was performed initially for infection screening, then to confirm *H. pylori* species by detecting the virulence gene (CagA). The gene encoding UreA produces 200-bp and 550-bp fragments of the CagA (Table 1). After being stained with ethidium bromide and examined under UV light, the amplified products were examined by 1.5% agarose gel electrophoresis.

### Genotyping of VDR

The SNPs for rs2228570 and rs3782905 were found using the polymerase chain reaction-restriction fragment length polymorphism (PCR-RFLP) technique. Table 1 displays the restriction enzymes, PCR settings, and primer sequences. Ethidium bromide staining was used to visualise the digested PCR fragments after they had been separated by 3% agarose gel electrophoresis. For the rs2228570 polymorphism, the fragments of 267 bp revealed homozygosity for the C allele, 204 bp and 63 bp fragments indicated homozygosity for the T allele. For the rs3782905 polymorphism, the fragments of 304 bp revealed homozygosity for the GG, and 223 bp and 81 bp revealed homozygosity for the C allele.

**Table 1:** Primer sequences, PCR conditions, and restriction enzymes

Primers	Sequences	PCR conditions	Restriction enzymes
UreA (F1)	5’-ATATTATGGAAGAAGCGAGAGC-3’	35 cycles of 94 °C for 1 min, 57 °C for 1 min, and 72 °C for 1 min 30 s.	--
UreA (R1)	5’- ATGGAAGTGTGAGCCGATTTG-3’		
UreA (F2)	5’- CATGAAGTGGGTATTGAAGC-3’		
UreA (R2)	5’-AAGTGTTGAGCCGATTTGAACCG-3’		
Cag A (F1)	5’- GGAACCCTAGTC AGTAATGGGTT-3’	35 cycles at 94 °C for 15 s, 55 °C for 30 s and72°C for 30 s	
CagA (R1)	5’- GCTTTAGCTTCTGATACCGCTTGA-3’		
CagA (F2)	5’-CCATTAACAATAATAATGGACTCAA-3’	35 cycles at 98 °C for 10 s 63 °C for 30 s and72°C for 30 s	
CagA (R2)	5’- AATTCTTGTTCCCTTGAAAGCCC-3’		
FokI rs2228570 (F)	5’-AGCTGGCCCTGGCACTGACTCTGGCTCT-3’	30 cycles of 95 ˚C for 45 s, 60 ˚C for 45 s, and 72 ˚C for 45 s	**BseG I**
FokI rs2228570 (R)	5’-GGTTAGATCGATATGTTTGA-3’		
BsmI rs3782905 (F)	5’-AAGACATGGTGTCTGCTTCA-3’	30 cycles of 95 ˚C for 45 s, 56 ˚C for 45 s, and 72 ˚C for 45 s	**HpyF3 I**
BsmI rs3782905 (R)	5’-GGTTAGATCGATATGTTTGA-3’		

### Statistical analysis

The raw data were entered and analyzed using SPSS v28.0 (IBM Corp., Armonk, NY, USA). Quantitative data were described using frequency and mean ± standard deviation (SD). The chi-square (χ²) test was used to assess the relationship between *H.pylori* infection susceptibility and genotype/allelic frequencies. Odds ratios (ORs) were calculated with a 95% confidence interval (CI). Differences in allele and genotype frequencies between patients and controls were evaluated using Fisher’s exact test. Statistical significance set at *P* < 0.05.

## Results

### Patients bassline characteristics

This study was performed on 300 patients, comprising 246 males (82.0%) with a mean age of 50.4 ± 8.6 years. Table 2 displays the individuals’ biochemical, clinical, and demographic details. Age and all biochemical indicators, with the exception of cholesterol and sodium, showed significant differences across the three groups in our investigation, but variations in smoking status and gender were statistically insignificant.

**Table 2:** The demographic, clinical, and biochemical characteristics of participants

Variables	Healthy control (*n* = 100)	LC patients (*n* = 100)	HCC patients (*n* = 100)	*P*-Value
Sociodemographic data				
Age	49.7 ± 4.1	46.7 ± 15.1	55.0 ± 6.7	< 0.001^*^
Gender (M/F)	72/28	91/9	83/17	1.00
Smoking (Yes/No)	59/41	77/23	79/21	0.199
Hematological parameters				
Hb (gm/dl)	13.5 ± 0.9	11.3 ± 2.3	10.4 ± 1.2	< 0.001^*^
WBCs (x103/mm3)	6.6 ± 1.8	4.3 ± 1.7	3.5 ± 1.2	< 0.001^*^
Platelets (x103/mm3)	266.9 ± 66.3	131.7 ± 66.1	107.4 ± 48.0	< 0.001^*^
Liver functions				
ALT (IU/L)	25.5 ± 8.0	49.8 ± 22.7	56.6 ± 26.4	< 0.001^*^
AST (IU/L)	27.3 ± 7.6	51.2 ± 23.9	55.6 ± 22.4	< 0.001^*^
Albumin (gm/dL)	4.4 ± 0.4	3.3 ± 0.5	3.0 ± 0.5	< 0.001^*^
T. protein (gm/dL)	7.1 ± 0.5	6.3 ± 0.7	5.7 ± 0.9	< 0.001^*^
Total bilirubin (mg/dL)	0.6 ± 0.2	1.5 ± 1.1	3.4 ± 2.8	< 0.001^*^
Direct bilirubin (mg/dL)	0.2 ± 0.1	0.7 ± 0.6	1.7 ± 1.0	< 0.001^*^
GGT (IU/L)	19.9 ± 6.5	50.7 ± 13.6	71.4 ± 21.5	< 0.001^*^
ALP (IU/L)	51.0 ± 14.4	83.9 ± 23.0	119.9 ± 35.4	< 0.001^*^
AFP (ng/mL)	5.1 ± 2.1	52.0 ± 78.4	377.4 ± 520.6	< 0.001^*^
Kidney functions				
Urea (mg/dL)	25.6 ± 4.3	32.8 ± 10.1	27.1 ± 5.5	< 0.001
Creatinine (mg/dL)	0.8 ± 0.2	0.9 ± 0.3	0.9 ± 0.2	^*^0.003
Uric Acid (mg/dL)	4.9 ± 2.4	5.8 ± 1.6	5.7 ± 2.3	^*^0.005
Electrolytes				
Sodium (Na) (mmol/L)	135.0 ± 3.3	134.2 ± 5.2	134.0 ± 4.4	0.232
Potassium (K) (mmol/L)	3.7 ± 0.5	4.0 ± 0.7	4.3 ± 0.3	< 0.001^*^
Lipid Profile				
Cholesterol (mg/dL)	119.4 ± 22.8	121.8 ± 38.8	± 37.8126.4	50.33
Triglyceride (TG) (mg/dL)	54.6 ± 27.9	75.7 ± 29.2	100.0 ± 45.9	< 0.001^*^
High-density lipoprotein (HDL) (mg/dL)	64.8 ± 21.5	55.3 ± 14.7	36.3 ± 10.2	< 0.001^*^
Low-density lipoprotein (LDL) (mg/dL)	59.5 ± 41.4	72.2 ± 32.5	39.1 ± 74.4	0.012^*^

Data represented as mean and standard deviation (SD)

*Hb* Hemoglobin, *WBCs* White blood cells, *ALT* Alanine aminotransferase, *AST* Aspartate amino transferase, *T. protein* Total Protein, *GGT* Gamma-glutamyl transferase, *ALP* Alkaline phosphatase, *AFP* Alphafetoprotein

^*^Significant difference at *P* < 0.05

### *H.pylori* prevalence among the studied groups

**Fig. 1:**
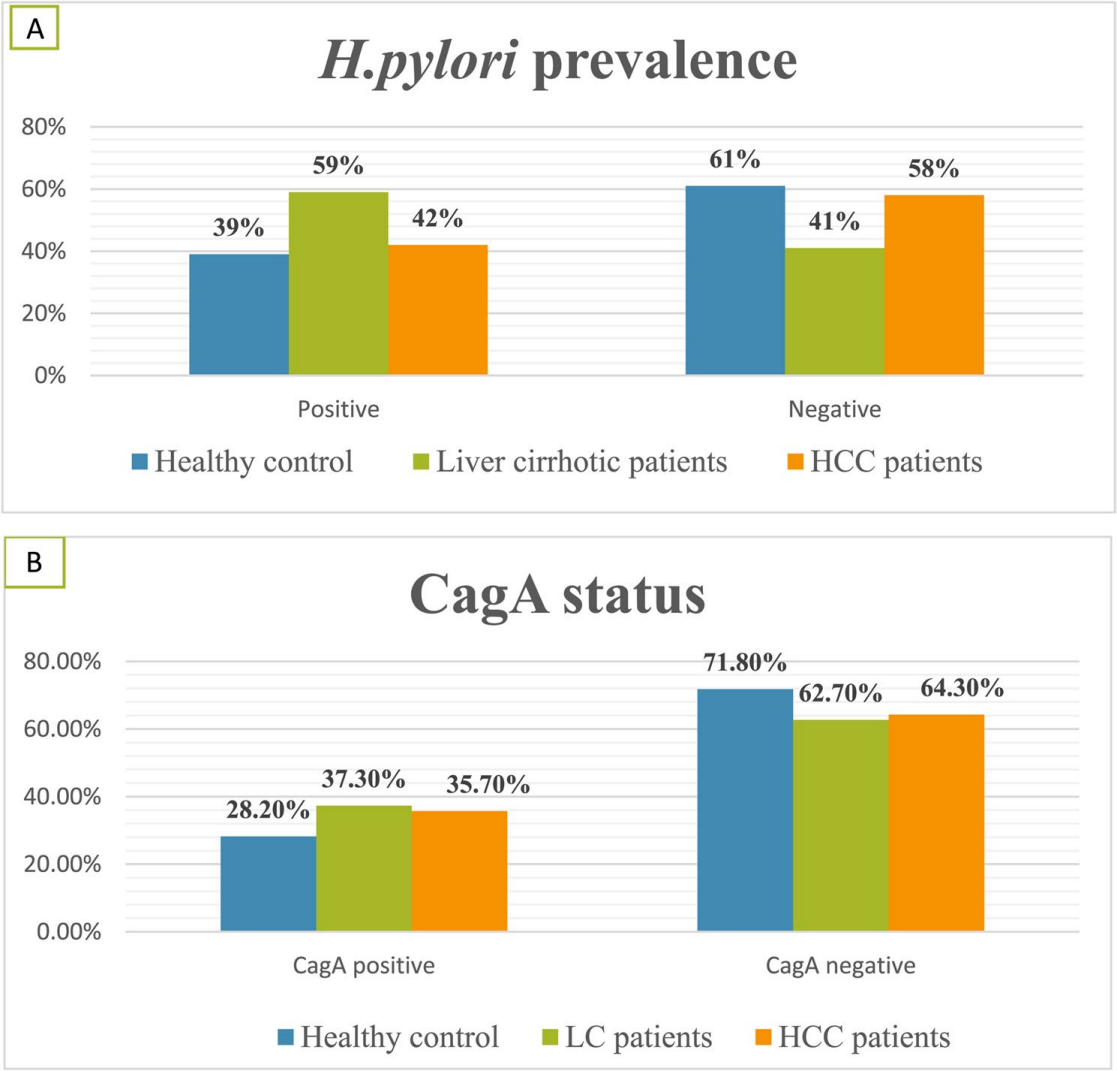
The distribution of **A**: *H.pylori*, **B**: *H.pylori CagA* status among different groups

Approximately half of our participants, 140/300 (46.7%), were found to have *H. pylori* infection. The prevalence of *H. pylori* infection among healthy controls, LC, and HCC patients were 39%, 59%, and 42%, respectively. With respect to the prevalence of onco-protein *CagA* gene in the positive samples, 28.2%, 37.3%, and 35.7% were identified as *CagA* positive among healthy controls, liver cirrhotic, and HCC patients, respectively (Fig. 1).

### Comparison of biochemical parameters and *H. Pylori* infection in liver cirrhosis and HCC patients

There was a significant difference in smoking status between LC patients with *H. pylori-*positive and *H. pylori-*negative (*P* = 0.002) when comparing sociodemographic data. On the other hand, there were significant differences in age, gender, and smoking status among HCC patients (*P* < 0.000, *P* = 0.002, and *P* = 0.008, respectively). In Table 3, the biochemical (liver, kidney, lipid, and electrolytes) and haematological associations with *H. pylori* status are displayed.

**Table 3:** Changes in sociodemographic and biochemical parameters with *H.pylori* infection

Variables	LC patients with H.pylori positive (*n* = 59)	LC patients with H.pylori negative (*n* = 41)	*P*-value	HCC patients with *H.pylori* positive (*n* = 42)	HCC patients with negative H.pylori (*n* = 58)	*P*-Value
Sociodemographic data						
Age	48.5 ± 15.3	44.0 ± 14.3	0.141	58.8 ± 4.1	52.3 ± 7.0	< 0.0001^*^
Gender (M/F)	54/5	37/4	0.252	25/17	58/0	0.002^*^
Smoking (Yes/No)	52/7	25/16	0.002^*^	39/3	40/18	0.008^*^
Hematological parameters						
Hb (gm/dl)	10.6 ± 2.6	12.4 ± 1.3	< 0.0001^*^	10.3 ± 2.2	10.6 ± 0.8	0.341
WBCS (x103/mm3)	4.0 ± 1.4	4.2 ± 1.8	0.471	3.2 ± 0.9	3.8 ± 1.1	0.004^*^
Platelets (x103/mm3)	81.9 ± 55.8	136.6 ± 52.7	< 0.0001^*^	67.7 ± 25.7	130.7 ± 97.0	0.0001^*^
Liver functions						
ALT (IU/L)	42.8 ± 29.9	50.7 ± 31.3	0.205	51.6 ± 26.5	20.2 ± 57.1	0.242
AST (IU/L)	73.1 ± 39.3	62.4 ± 29.7	0.144	53.8 ± 22.7	62.1 ± 29.9	0.134
Albumin (gm/dL)	2.7 ± 0.5	3.1 ± 0.7	0.001^*^	2.7 ± 0.3	3.1 ± 0.5	< 0.0001^*^
T. protein (gm/dL)	6.6 ± 0.7	7.1 ± 0.5	0.0002^*^	6.1 ± 1.1	5.4 ± 0.8	0.0004^*^
Total bilirubin (mg/dL)	1.9 ± 0.7	1.2 ± 1.4	0.001^*^	3.5 ± 2.2	3.3 ± 1.7	0.609
Direct bilirubin (mg/dL)	1.0 ± 0.7	0.6 ± 0.7	0.006^*^	1.9 ± 0.5	1.3 ± 0.3	< 0.0001^*^
GGT (IU/L)	55.9 ± 12.2	52.5 ± 14.8	0.212	75.6 ± 19.4	66.8 ± 23.7	0.05^*^
ALP (IU/L)	91.3 ± 18.9	88.8 ± 25.4	0.574	122.4 ± 36.6	116.5 ± 34.8	0.415
AFP (ng/mL)	61.7 ± 62.6	46.3 ± 89.4	0.313	387.6 ± 528.3	367.2 ± 512.9	0.847
Kidney functions						
Urea (mg/dL)	32.7 ± 9.0	32.9 ± 11.4	0.922	25.2 ± 7.2	3.2 ± 28.2	0.006^*^
Creatinine (mg/dL)	0.8 ± 0.2	1.0 ± 0.2	< 0.0001^*^	0.8 ± 0.2	1.0 ± 0.2	< 0.0001^*^
Uric Acid(mg/dL)	5.7 ± 1.3	5.9 ± 1.9	0.533	4.9 ± 1.8	6.2 ± 2.4	0.004^*^
Electrolytes						
Sodium (Na) (mmol/L)	132.6 ± 5.5	137.1 ± 2.9	< 0.0001^*^	132.2 ± 4.2	134.0 ± 4.4	0.042^*^
Potassium (K) (mmol/L)	4.0 ± 0.7	4.1 ± 0.8	0.509	4.2 ± 0.4	4.3 ± 0.2	0.104
Lipid profile						
Cholesterol (mg/dL)	115.4 ± 37.5	132.3 ± 38.6	0.031^*^	149.8 ± 41.2	109.7 ± 23.8	< 0.0001^*^
Triglyceride (TG) (mg/dL)	78.5 ± 32.7	71.3 ± 21.6	0.220	92.8 ± 44.5	105.3 ± 46.2	0.178
High-density lipoprotein (HDL) (mg/dL)	63.4 ± 71.3	43.0 ± 13.1	0.074^*^	39.2 ± 10.6	34.3 ± 9.4	0.017^*^
Low-density lipoprotein (LDL) (mg/dL)	66.9 ± 30.2	81.1 ± 34.2	0.031^*^	102.6 ± 43.0	54.3 ± 18.3	< 0.0001^*^

Data represented as mean and standard deviation (SD)

*Hb* Hemoglobin, *WBCs* White blood cells, *ALT* Alanine aminotransferase, *AST* Aspartate amino transferase, *T. protein* Total Protein, *GGT* Gamma-glutamyl transferase, *ALP* Alkaline phosphatase, *AFP* Alphafetoprotein

^*^Significant difference at *P* < 0.05

### Diagnostic performance for *H. pylori* prevalence

**Fig. 2:**
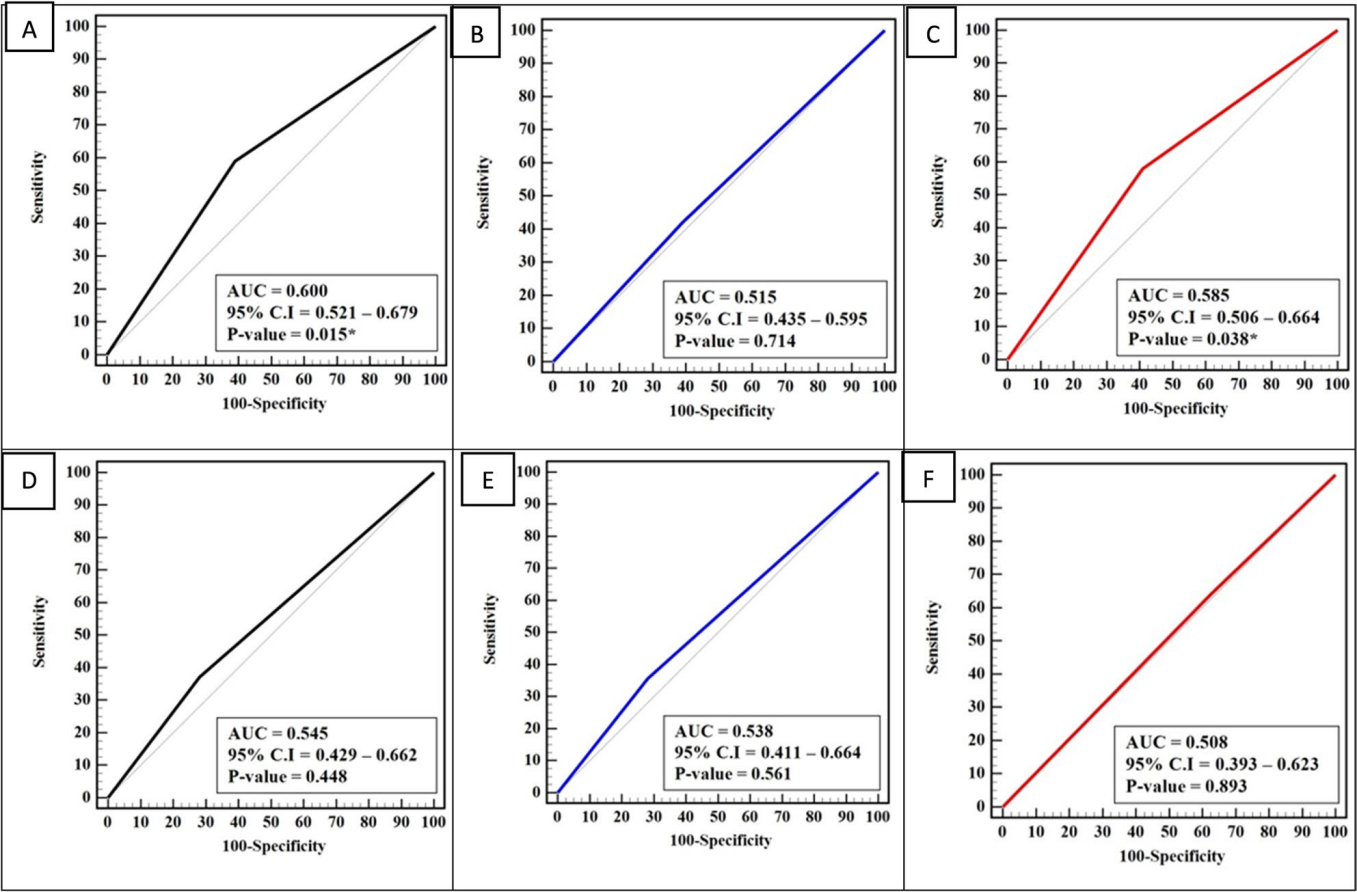
ROC Curve Analysis for Biomarker Diagnostic Performance of *H.pylori* and CagA status; **A** discriminate H.pylori infected cirrhotic patients (*n* = 100) from infected control (*n* = 100); **B** discriminate H.pylori infected HCC patients (*n* = 100) from infected control (*n* = 100); **C** discriminate H.pylori infected HCC patients (*n* = 100) from infected cirrhotic patients (*n* = 100); **D** discriminate cirrhotic patients infected with CagA strain from control infected with CagA strain; **E** discriminate HCC patients infected with CagA strain from control infected with CagA strain; and **F** discriminate HCC patients infected with CagA strain from cirrhotic patients infected with CagA strain

The receiver operating characteristic (ROC) curve analysis demonstrated modest discriminatory ability for the biomarker in distinguishing cirrhotic patients from healthy controls, with an AUC of 0.600 (95% CI: 0.521–0.679; *P* = 0.015), yielding 59% sensitivity, 61% specificity, and 60% accuracy. In contrast, differentiation of HCC patients from healthy controls showed poor performance (AUC = 0.515; 95% CI: 0.435–0.595; *P* = 0.714), with 42% sensitivity and 51.5% accuracy. Between HCC and cirrhotic patients, the AUC was 0.585 (95% CI: 0.506–0.664; *P* = 0.038), but metrics were low (42% sensitivity, 41% specificity, 41.5% accuracy) (Fig. 2A-C).

ROC curve analysis revealed poor discriminatory performance of *H .pylori* prevalence as a biomarker across all groups. Distinguishing *H.pylori* infected cirrhotic patients (*n* = 59) from infected controls (*n* = 39) yielded an AUC of 0.545 (95% CI: 0.429–0.662; *P* = 0.448), with 37.29% sensitivity, 71.79% specificity, and 51.02% accuracy. *H.pylori* infected HCC patients (*n* = 42) versus infected controls showed an AUC of 0.538 (95% CI: 0.411–0.664; *P* = 0.561), with 35.71% sensitivity and 53.09% accuracy. Differentiation between *H.pylori* infected HCC and cirrhotic patients was negligible (AUC = 0.508; 95% CI: 0.393–0.623; *P* = 0.893), achieving only 35.71% sensitivity, 62.71% specificity, and 51.49% accuracy (Fig. 2D-F).

### VDR SNPs genotype and allele frequency in the studied groups

**Fig. 3:**
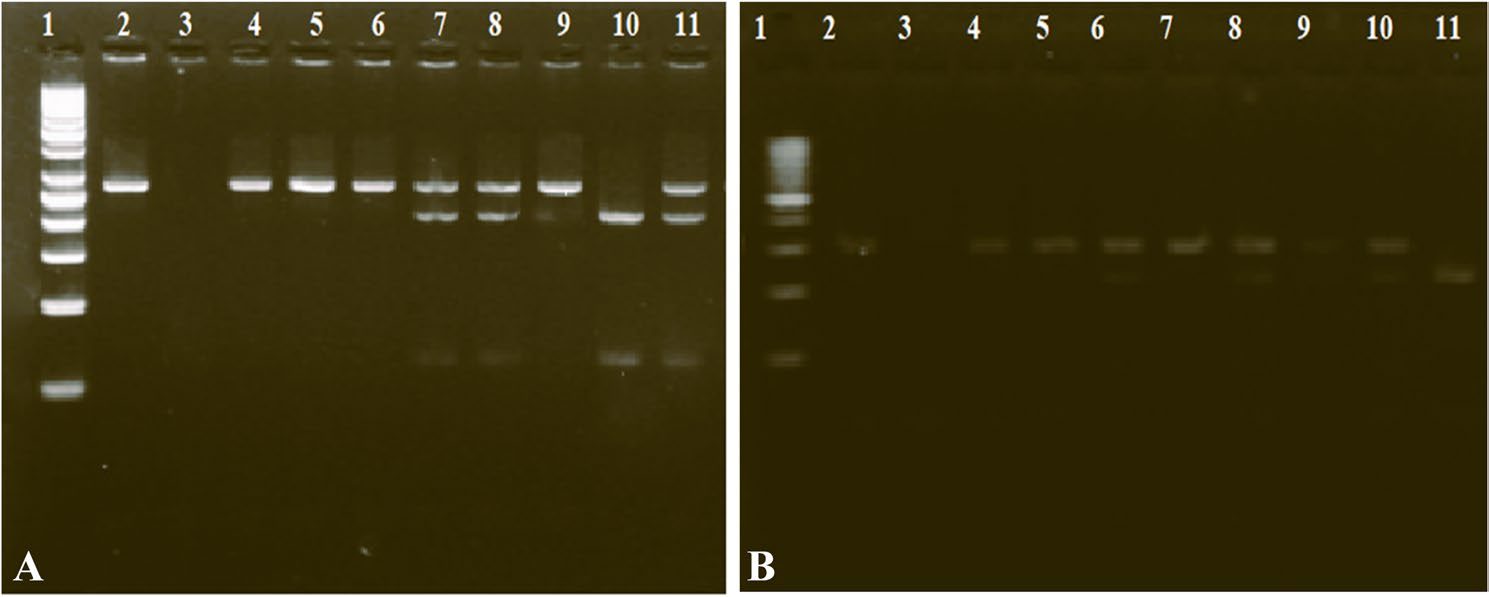
**A** Gel electrophoresis for the rs2228570 polymorphism of the VDR gene. The 267 bp bands correspond to wild homozygous CC, which produced one fragment, while the 267, 204, and 63 bp bands correspond to heterozygous CT that produced three fragments. The 204 and 63 bp correspond to the mutant homozygous TT. Lane 1: 50 bp ladder, Lane 2: Positive control before digestion, Lane 3: Negative control (Nuclease-free water), Lanes: (4, 5, 6) were CC genotype; Lanes (7, 8, 9, 11) were CT genotype, and Lane (10) was TT genotype. **B** Agarose gel electrophoresis for the rs3782905 polymorphism of the VDR gene. The 304 bp bands correspond to wild homozygous GG, which produced one fragment, while the 304, 223, and 81 bp bands correspond to heterozygous GC that produced three fragments. The 223 and 81 bp correspond to the mutant homozygous CC. Lane 1: 100 bp ladder, Lane 2: Positive control before digestion, Lane 3: Negative control (Nuclease-free water), Lanes: (4, 5, 7) were GG genotype; Lanes (6, 8, 9, 10) were GC genotype, and Lane (11) was CC genotype. The 81 bp was invisible in the gel due to its fast migration speed

For variant analysis, the obtained samples were analyzed for SNPs at the VDR FokI and BsmI genes (rs2228570 and rs3782905). At rs2228570, the genotype frequencies were 34.0%, 48.0%, and 18.0% for CC, CT, and TT, respectively, in the healthy control group. In LC patients, CC, CT, and TT genotype frequencies were 54.0%, 26.0%, and 20.0%, respectively, while in HCC patients were 46.0%, 33.0%, and 21.0% respectively (Table 4; Fig. 3A). A high percentage of homozygosity (CC) was recorded at this locus in LC patients and HCC patients, and the CT genotype percentage was found to be the lowest. The differences in the CC and CT genotype frequencies were statistically significant (*P* = 0.004 and *P* = 0.001, respectively) between LC patients and healthy controls, and the differences between HCC patients and healthy controls were statistically significant (*P* = 0.084 and *P* = 0.022, respectively). The CT genotype might be considered a protective factor for LC and HCC (OR = 2.63, 95% CI = 1.449–4.761, *P* = 0.001) and (OR = 1.96, 95% CI = 1.104–3.485, *P* = 0.022), respectively. The C and T allelic frequencies among the three studied groups were insignificantly different.

At rs3782905, GG, GC, and CC genotype frequencies were 34.0%, 48.0%, and 18.0%, respectively, in the healthy control group. In LC patients, CC, CT, and TT genotype frequencies were 54.0%, 26.0%, and 20.0%, respectively, while in HCC patients were 46.0%, 33.0%, and 21.0% respectively (Table 4, Fig. 3B). A high percentage of homozygosity (CC) was recorded at this locus in LC patients and HCC patients, and the GG genotype percentage was found to be the lowest. The differences in the CC and GG genotype frequencies were statistically significant (*P* < 0.0001) between LC patients and healthy controls, as well as the differences between HCC patients and healthy controls (*P* < 0.0001). The CC genotype might be considered a risk factor for LC and HCC (OR = 4.16, 95% CI = 2.223–7.798, *P* < 0.0001) and (OR = 1.79, 95% CI = 0.940–3.435, *P* = 0.076), respectively. On the other hand, the GG might be considered a protective factor from LC and HCC (OR = 4.44, 95% CI = 2.351–8.374, *P* < 0.0001) and (OR = 3.48, 95% CI = 1.896–6.405, *P* = 0.0001), respectively. The G and C allelic frequencies showed a significant difference between the three studied groups. The G allele was significantly higher in LC and HCC patients than in healthy controls, while the C allele was lower in LC and HCC patients than in healthy controls (Table 4).

**Table 4:** Comparison between the studied groups according to *FokI* and *BsmI* genotypes and allelic frequency

SNP	Healthy Control (*N* = 100)	LC patients (*N* = 100)	HCC patients (*N* = 100)	LC vs. Healthy controls		HCC patient’s vs. Healthy controls		LC vs. HCC patients	
				OR (95% CI)	*P* value	OR (95% CI)	*P* value	OR (95% CI)	*P* value
VDR (FokI rs2228570) Allele and genotypes									
C	116 (58.0%)	134 (67.0%)	124 (62.0%)	0.68 (0.383–1.209)	0.189	0.85 (0.480–1.491)	0.564	1.24 (0.697–2.223)	0.460
T	84 (42.0%)	66 (33.0%)	76 (38.0%)	1.47 (0.827–2.615)	0.189	1.18 (0.671–2.082)	0.564	0.80 (0.449–1.436)	0.460
CC	34 (34.0%)	54 (54.0%)	46 (46.0%)	0.44 (0.248–0.777)	0.004^*^	0.60 (0.342–1.070)	0.084^*^	1.38 (0.790–2.403)	0.258
CT	48 (48.0%)	26 (26.0%)	33 (33.0%)	2.63 (1.449- 4.761)	0.001^*^	1.96 (1.104–3.485)	0.022^*^	1.34 (0.725–2.473)	0.350
TT	18 (18.0%)	20 (20.0%)	21 (21.0%)	1.14 (0.561–2.310)	0.719	1.21 (0.601–2.442)	0.593	1.06 (0.535–2.113)	0.861
CT and TT	66 (66.0%)	46 (46.0%)	54 (54.0%)	2.28 (1.288–4.033)	0.004^*^	1.72 (0.973–3.045)	0.062^*^	1.32 (0.759–2.308)	0.323
VDR (BsmI rs3782905) Allele and genotypes									
G	69 (34.0%)	132 (66.0%)	107 (53.0%)	3.77 (2.099–6.765)	< 0.0001^*^	0.58 (0.328- 1.028)	0.062^*^	0.58 (0.328- 1.028)	0.062^*^
C	131(66.0%)	68 (34.0%)	93 (47.0%)	0.27 (0.148–0.476)	< 0.0001^*^	1.72 (0.973–3.045)	0.062^*^	1.72 (0.973–3.045)	0.062^*^
GG	51 (51.0%)	19 (19.0%)	23 (23.0%)	4.44 (2.351–8.374)	< 0.0001^*^	3.48 (1.896–6.405)	0.0001^*^	1.27 (0.643–2.521)	0.488
GC	29 (29.0%)	30 (30.0%)	45 (45.0%)	1.05 (0.571–1.927)	0.877	2.0 (1.116–3.594)	0.0199^*^	1.91 (1.067–3.415)	0.029^*^
CC	20 (20.0%)	51 (51.0%)	32 (32.0%)	4.16 (2.223–7.798)	< 0.0001^*^	1.79 (0.940–3.435)	0.076^*^	0.43 (0.242- 0.769)	0.004^*^
GC and CC	49 (49.0%)	81 (81.0%)	76 (76.0%)	4.44 (2.351–8.374)	< 0.0001^*^	3.29 (1.802–6.027)	0.0001^*^	0.74 (0.377- 1.464)	0.390

^*^Significant difference at *P* < 0.05

### VDR SNPs genotype and allele frequency with *H.pylori* infection

*H. pylori-*infected and uninfected patients differed significantly, according to an analysis of the genotypic and allelic distributions of the FokI and BsmI polymorphisms in the VDR gene (Table 5). Regarding the FokI polymorphism, LC patients with an *H. pylori* infection had a higher frequency of the CC genotype than those without an infection (62.7% vs. 41.4%, respectively), and an OR of 2.45 indicates a risk effect of this genotype. However, compared to the H. pylori-positive group, the TT genotype was more prevalent in the LC patients in the *H. pylori*-negative group (36.6% vs. 8.5%, respectively). In contrast, HCC patients with *H. pylori* infection had a higher frequency of the CT genotype than *H. pylori* uninfected (62.0% vs. 12.1%, respectively), and an OR of 11.96 indicates a risk effect of this genotype. The TT and CC genotypes, on the other hand, were more prevalent in the HCC patients with *H. pylori*-uninfected than in *H. pylori*-infected patients. While there was no significant difference in the allelic distribution between HCC patients with and without H. pylori infection, the allelic frequencies of the C and T alleles showed a significant increase in the C allele in LC patients with *H. pylori*-infected than *H. pylori*-uninfected, with an OR of 3.09.

Regarding the *BsmI* polymorphism, LC patients with an *H. pylori-positive status* had a higher frequency of the GC genotype than *H. pylori-*negative (40.7% vs. 12.2%, respectively), and an OR of 5.1 indicates a risk effect of this genotype. Also, the CC genotype was higher in LC patients with *H. pylori*-positive than *H. pylori-*negative (32.2% vs. 2.4%, respectively), and an OR of 23.1 implies a risk effect of this genotype. On the other hand, LC patients with *H. pylori*-negative status had a higher frequency of the GG genotype than those with *H. pylori*-positive status (85.4% vs. 27.1%, respectively). When comparing LC patients with an *H. pylori* infection to those without, the allelic frequencies of the G and C alleles revealed a significant increase in the C allele, and an OR of 11.4 indicates a risk effect of this variant. However, there is no significant difference between HCC patients with and without an *H. pylori* infection in terms of *BsmI* genotypic and allelic frequencies (Table 5).

**Table 5:** Genotypic and allelic frequencies and significant difference of VDR gene polymorphisms in *H. pylori*-infected and uninfected patients

SNP	LC patients with H.pylori positive (*n* = 59)	LC patients with H.pylori negative (*n* = 41)	OR (95% CI)	*P* value	HCC patients with *H.pylori* positive (*n* = 42)	HCC patients with *H.pylori* negative (*n* = 58)	OR (95% CI)	*P* value
VDR (FokI rs2228570) Allele and genotypes								
C	91 (77.1%)	43 (52.4%)	3.09 (1.681–5.682)	0.0003^*^	52 (61.9%)	73 (62.9%)	0.96 (0.541–1.699)	0.884
T	27 (22.9%)	39 (47.6%)	0.32 (0.176–0.595)	0.0003^*^	32 (38.1%)	43 (37.1%)	1.04 (0.589–1.850)	0.884
CC	37 (62.7%)	17 (41.4%)	2.45 (1.387–4.328)	0.002^*^	13 (31.0%)	33 (56.9%)	0.34 (0.189–0.605)	0.0003^*^
CT	17 (28.8%)	9 (22.0%)	1.45 (0.763–2.748)	0.257	26 (62.0%)	7 (12.1%)	11.96 (5.790- 24.725)	< 0.0001^*^
TT	5 (8.5%)	15 (36.6%)	0.19 (0.085–0.421)	< 0.0001^*^	3 (7.0%)	18 (31.0%)	0.17 (0.069–0.403)	0.0001^*^
CT and TT	23 (37.3%)	24 (58.6%)	0.41 (0.231–0.721)	0.002^*^	29 (69.0%)	25 (43.1%)	2.95 (1.652–5.269)	0.0003^*^
VDR (BsmI rs3782905) Allele and genotypes								
G	56 (47.5%)	75 (91.4%)	0.09 (0.042–0.201)	< 0.0001^*^	38 (45.2%)	53 (45.7%)	0.96 (0.551–1.676)	0.887
C	62 (52.5%)	7 (8.6%)	11.4 (5.177–25.109)	< 0.0001*	46 (54.8%)	63 (54.3%)	1.04 (0.597–1.817)	0.887
GG	16 (27.1%)	35 (85.4%)	0.06 (0.029–0.123)	< 0.0001*	9 (21.4%)	14 (24.1%)	0.84 (0.433–1.637)	0.612
GC	24 (40.7%)	5 (12.2%)	5.1 (2.473–10.500)	< 0.0001*	20 (47.6%)	25 (43.1%)	1.22 (0.701–2.137)	0.478
CC	19 (32.2%)	1 (2.4%)	23.1 (5.346–99.459)	< 0.0001*	13 (31.0%)	19 (32.8%)	0.91 (0.503–1.653)	0.762
GC and CC	43 (72.9%)	6 (14.6%)	15.3 (7.575–30.989)	< 0.0001*	33 (78.6%)	44 (75.9%)	1.19 (0.611–2.309)	0.612

^*^Significant difference at *P* < 0.05

### VDR SNPs genotype and allele frequency with *H.pylori* CagA status

Table 6 summarises the genotypic and allelic distributions of the *FokI* and *BsmI* polymorphisms in the VDR gene concerning *H. pylori CagA* status. Regarding the *FokI* polymorphism, LC patients with *H. pylori-CagA-*positive had a higher frequency of the TT genotype than those with *H. pylori CagA-*negative patients (13.6% vs. 5.4%, respectively), and an OR of 3.1 indicates that this genotype has a risk effect. Additionally, HCC patients with *H. pylori-CagA-*positive had a higher frequency of the TT genotype than those with *H. pylori-CagA* negative (13.3% vs. 3.7%, respectively), and an OR of 3.6 indicates that this genotype has a risk effect.

Regarding the BsmI polymorphism, there was no significant difference in the allelic distribution between LC, HCC patients with and without *H. pylori* infection. The GC genotype was more common in HCC patients with *H. pylori-CagA*-negative than in the *H. pylori-CagA*-positive (60.0% vs. 40.8%, respectively), and an OR of 2.2 indicates a risk effect of this genotype. On the other hand, the CC genotype was more common in the LC patients with *H. pylori-CagA*-negative than in the *H. pylori-CagA-*positive (37.8% vs. 22.7%, respectively).

**Table 6:** Genotypic and allelic frequencies and significant difference of VDR gene polymorphisms in *H. pylori-CagA* positive and *H. pylori-CagA* negative patients

SNP	LC patients with *H.pylori* Cag A positive (*n* = 22)	LC patients with *H.pylori* Cag A negative (*n* = 37)	OR (95% CI)	*P* value	HCC patients with *H.pylori* Cag A positive (*n* = 15)	HCC patients with *H.pylori* Cag A negative (*n* = 27)	OR (95% CI)	*P* value
VDR (FokI rs2228570) Allele and genotypes								
C	32 (72.7%)	59 (79.7%)	0.68 (0.349–1.307)	0.244	17 (56.7%)	35 (64.8%)	0.71 (0.403–1.263)	0.247
T	12 (27.3%)	15 (20.3%)	1.48 (0.765–2.861)	0.244	13 (44.3%)	19 (35.2%)	1.5 (0.825–2.579)	0.194
CC	13 (59.1%)	24 (64.9%)	0.77 (0.437–1.374)	0.386	4 (26.7%)	9 (33.3%)	0.75 (0.409–1.378)	0.355
CT	6 (27.3%)	11 (29.7%)	0.86 (0.467–1.596)	0.639	9 (60.0%)	17 (63.0%)	0.88 (0.498–1.558)	0.663
TT	3 (13.6%)	2 (5.4%)	3.1 (1.069–8.945)	0.037^*^	2 (13.3%)	1 (3.7%)	3.6 (1.127–11.413)	0.031^*^
CT and TT	9 (40.9%)	13 (35.1%)	1.3 (0.728–2.288)	0.383	11 (73.3%)	18 (66.7%)	1.3 (0.726–2.444)	0.355
VDR (BsmI rs3782905) Allele and genotypes								
G	24 (54.5%)	32 (50.0%)	1.2 (0.701–2.131)	0.479	15 (50.0%)	23 (42.6%)	1.3 (0.759–2.314)	0.321
C	20 (45.5%)	32 (50.0%)	0.82 (0.469–1.426)	0.479	15 (50.0%)	31 (57.4%)	0.75 (0.432–1.317)	0.321
GG	7 (31.8%)	9 (24.4%)	1.5 (0.799–2.776)	0.209	3 (20.0%)	6 (22.2%)	0.89 (0.449–1.752)	0.729
GC	10 (45.5%)	14 (37.8%)	1.4 (0.791–2.442)	0.252	9 (60.0%)	11 (40.8%)	2.2 (1.227–3.797)	0.007^*^
CC	5 (22.7%)	14 (37.8%)	0.49 (0.263–0.903)	0.022^*^	3 (20.0%)	10 (37.0%)	0.43 (0.225–0.804)	0.009^*^
GC and CC	15 (68.2%)	28 (75.6%)	0.67 (0.360–1.250)	0.209	12 (80.0%)	22 (77.8%)	1.1 (0.571–2.229)	0.729

^*^Significant difference at *P* < 0.05

## Discussion

Vitamin D Receptor (VDR) expression in gastric epithelia was assumed to have improved as a result of the *H. pylori* infection. This resulted in immune modulators that effectively combat this pathogen [22]. This study was aimed at investigating the prevalence of *H. pylori* infection in liver disease patients and the relationship between vitamin D receptor gene variants and *H. pylori* infection and HCC risk in liver disease patients from Egypt.

The study found that elderly patients had a significantly higher overall HCC risk than both LC patients and healthy controls (*P* < 0.001). In terms of biochemical parameters, the three groups under study showed significant differences in Hb, total leucocyte count, platelets, ALT, AST, albumin, T. protein, total bilirubin, direct bilirubin, GGT, ALP, urea, creatinine, uric acid, AFP, triglycerides, HDL, and LDL levels. Turshudzhyan and Wu [23], and El-masry et al. [24] found that patients with HCC had higher serum levels of ALT, AST, bilirubin, creatinine, and AFP than patients with chronic liver disease and healthy controls.

In our study, 59% of individuals with liver cirrhosis had an *H. pylori*-positive test result. This finding aligns with several studies, including Pati et al. [25] in India, which found that 57.4% of 864 liver cirrhosis patients had an *H.Pylori* infection, and Abdel-Razik et al. [26] in Egypt, which analysed data from 558 cirrhotic patients who had esophagogastroduodenoscopy (EGD) and discovered *H. pylori* infection in 51.6% of the patients. 42% of HCC patients had an *H. pylori* infection. Mekonnen et al. [27] discovered *H. pylori* in 61.7% of HCC patients, while Yousif et al. [28] found *H. pylori* in 76.7% of HCC patients. This prevalence was lower than that of the earlier studies. The cagA gene was found in 28.2%, 37.3%, and 35.7% of the healthy control, LC, and HCC patients, respectively, according to our study. Nonetheless, several studies have revealed varying cagA gene percentages in other countries [29–31].

In this study, the ROC analysis revealed modest discriminatory power of the biomarker for separating cirrhotic patients from healthy controls (AUC 0.600, *P* = 0.015), yet failed to effectively distinguish HCC cases from either group, with AUC values near chance level (0.515 and 0.585). These results suggest the biomarker captures some cirrhosis-related pathological changes but lacks specificity for the malignant transformation in HCC. The statistically significant p-values for cirrhosis vs. controls and HCC vs. cirrhosis comparisons indicate non-random discrimination, though low sensitivity (42–59%) and accuracy (41.5–60%) preclude standalone clinical use.

ROC curve analysis showed negligible discriminatory performance of *H.pylori* prevalence as a biomarker across all groups, with AUC values hovering near chance level (0.508–0.545) and non-significant p-values, reflecting very low sensitivity (35.71–37.29%) despite moderate specificity. The biomarker’s failure to distinguish *H. pylori-*infected cirrhotic patients from infected controls (AUC 0.545, *p* = 0.448), infected HCC from infected controls (AUC 0.538, *P* = 0.561), or infected HCC from infected cirrhosis (AUC 0.508, *P* = 0.893) indicates it does not capture disease-specific pathological signals in this study. Accuracies around 51–53% are indistinguishable from random classification, and low sensitivity severely limits its ability to detect true positives.

We found that older HCC patients were most frequently reported to have an *H. pylori* infection. According to Taylor et al. [32], this could be explained by the fact that as individuals age, the prevalence of infections in the population rises. *H. pylori* was found to significantly reduce haemoglobin, white blood cells, and platelets in the present study. According to Mwafy et al. [33], haematological alterations are associated with *H. pylori* infection, and this conclusion is consistent with their findings. Additionally, compared to patients who are not infected, *H. pylori*-infected patients have significantly lower levels of albumin, total protein, creatinine, uric acid, sodium, and LDL. This is in line with the findings of Liu et al. [34], who found that *H. Pylori* infection dramatically impacted nutritional metabolism by being associated with decreased serum albumin levels and a lower albumin/globulin ratio. In contrast, patients with *H. pylori* infection had significantly higher levels of direct bilirubin and HDL than those without the infection.

According to the current study, both LC and HCC patients had a noticeably high frequency of CC genotypes in *FokI* (rs2228570). Thus, the CC genotype may be linked to a little increase in the risk of liver cirrhosis in addition to being a twofold risk factor for HCC. On the other hand, the CT genotype might offer some protection against LC and HCC. This is in line with the results of Tsounis et al. [35], who discovered an association between the development of cirrhosis and homozygosity for the dominant phenotype of *FokI* variants. According to this study, the VDR gene polymorphism at *BsmI* (rs3782905) showed a substantial incidence of the CC genotype in patients with liver cirrhosis and HCC. This implies that the CC genotype may be a four-fold increased risk factor for LC and a two-fold increased risk factor for HCC compared to healthy controls. While the GC genotype increases the risk for the prognosis of LC to HCC, the GG genotype may be protective against both LC and HCC. These results were similar to those of El-masry et al. [24].

In the current study, there was a significant variation in the genotypic distribution of the *FokI* and *BsmI* SNPs between the *H. pylori*-infected and -uninfected samples. LC patients with *H. pylori*-positive (rs2228570) had a considerably higher prevalence of the CC genotype than LC patients with *H. pylori*-negative (rs2228570). This indicates that CC genotypes may be over two times as likely to be a risk factor for *H. pylori* infection in individuals with liver cirrhosis. The CT genotype may be 11 times more likely to be linked to *H. pylori* infection in HCC patients than in *H. pylori*-negative patients; however, *H. pylori*-positive HCC patients had a higher CT genotype than *H. pylori*-negative patients. Three times as many LC patients with the C allele may be at risk for *H. pylori*-positive conditions as those with *H. pylori*-negative conditions. The results of this study were consistent with those of Mohamed et al. [36], who found that the *H. pylori* positive group had the highest prevalence of CT and CC, whereas the *H. pylori* negative group had a higher TT.

In the present study, the *BsmI* polymorphism, the frequency of the GC and CC genotypes were higher in LC patients with *H. pylori*-infected than uninfected. The GC genotype might be a risk factor for *H.pylori* infection in LC patients more five times than LC patients with *H.pylori*-negative and the CC genotype might be a risk factor for *H.pylori* infection in LC patients more 23 times than LC patients with *H.pylori*-negative. The allelic frequencies showed a significant increase in C allele in LC patients with *H. pylori*-positive than *H. pylori* negative, suggesting that C allele might be a risk factor for *H.pylori* infection in LC patients more 11 times than LC patients with *H.pylori*-negative.

The rs2228570 for the CagA strain revealed a significant difference between *H. pylori*-CagA positive and *H. pylori* CagA negative. Patients with LC and HCC who had the TT genotype were more likely to have *H. pylori*-CagA positive than those who had the CagA negative. This implies that the TT genotype may be a risk factor for *H. pylori*-CagA positive in LC and HCC patients more than three times the TT genotype. *H. pylori*-CagA positive and *H. pylori* CagA negative were significantly different according to rs3782905. *H. pylori*-CagA positive patients had a higher GC genotype *than H. pylori*-CagA negative patients, indicating that the GC genotype may be a risk factor for *H. pylori*-CagA positive patients more than twice as much as *H. pylori*-CagA negative patients. On the other hand, LC patients with *H. pylori*-CagA-negative status had a higher prevalence of the CC genotype than those with *H. pylori*-CagA-positive status.

## Conclusion

Our results show a strong correlation between *H. pylori* infection and liver disorders, with a high prevalence of *H. pylori* infection among LC and HCC patients. A significant association between biochemical markers and *H. pylori* infection were identified. Regarding genetic predisposition, we discovered a potential correlation between the *FokI* and *BsmI* SNPs and *H. pylori* infection and virulence strain. Future randomized clinical trials and population-based research from wider geographic areas are necessary to assess the results and further explore this topic.

## Data Availability

No datasets were generated or analysed during the current study.

## Abbreviations

ACLD: Advanced-stage concurrent liver disease
AFP: Alpha-fetoprotein
EGD: Esophagogastroduodenoscopy
*H. pylori*: Helicobacter pylori
HCC: Hepatocellular carcinoma
LC: Liver cirrhosis
PCR: Polymerase chain reaction
RFLP-PCR: Restriction fragment length polymorphism-Polymerase chain reaction
VDRs: Vitamin D receptors
WHO: World Health Organization
